# College Announcements Received

**Published:** 1858-09

**Authors:** 


### COLLEGE ANNOUNCEMENTS RECEIVED.

From the "Medical College" of the State of South Carolina, Charleston—216 students and 42 graduates at the last session—course opens the second Monday in November and terminates the first Saturday in March.

"Memphis Medical College," Memphis, Tennessee—44 students and 18 graduates at the last course—session commences the 1st of Nov. and ends the 1st of March.

"College of Physicians and Surgeons of the Iowa State University" (location not stated,) at Keokuk we believe, 75 students and 20 graduates, session commences the 4th of Nov. and continues four months.

"Oglethorpe Medical College", Savannah, Geo.,—30 students and 11 graduates—the course commences on the 18th of October and closes the first week in March.

And last, though not least, the "Report and announcement" of our venerated Alma Mater, the time honored "Medical Department of the University of Pennsylvania," from which we learn that "the number of students in attendance upon the past course of Lectures was 435, and the number of graduates for the year was 145. The regular lectures of the coming session, 1858–59, will commence on Monday October 11, and be continued without interruption until the middle of March ensuing."

